# Clinical characteristics and therapeutic direction of HER2 low-expression breast cancer

**DOI:** 10.3389/fonc.2025.1484103

**Published:** 2025-02-27

**Authors:** Linlin Zhou, Yinghui Zhang, Jiayu Zhang, Hongyu Wang, Bozhi Zhao, Yixin Cai, Yuansong Qu, Xinxin Li, Dongwei Zhang

**Affiliations:** Department of Breast Surgery, The Second Affiliated Hospital of Harbin Medical University, Harbin, Heilongjiang, China

**Keywords:** HER2 low-expression breast cancer, HER2, clinical features, advanced treatment, targeted therapy

## Abstract

Human epidermal growth factor receptor 2 (HER2) is one of the oncogenic drivers of breast cancer and is often used as a definitive therapeutic marker for breast cancer. This has led to significant improvements in both targeted therapy and prognosis for HER2-targeted breast cancer. Due to the differences in HER2 gene and protein expression levels, they are clinically classified into HER2 zero-expression breast cancer, low-expression breast cancer and high-expression breast cancer. Among them, HER2 low-expression is considered a special expression state, which is insensitive to conventional anti-HER2 therapy and has a poorer prognosis and thus has received attention from researchers. Some studies demonstrate that patients with HER2 low-expression can benefit from antibody-drug conjugates (ADC). Several studies are currently exploring the efficacy of various ADC drugs in breast cancer with HER2 low-expression, opening up new treatment avenues for patients with HER2 low-expression breast cancer. This review aims to summarize the clinical features of HER2 low-expression breast cancer and the recent advances in its therapeutic agents.

## 1 Introduction

The latest data from the International Agency for Research on Cancer (IARC) suggests that by 2020, breast cancer has been one of the most common types of cancer worldwide and one of the leading causes of cancer death in women. More than 20,000 new cases of breast cancer have been diagnosed globally and nearly 266,850,000 people have already died from breast cancer in 2020 (1). Currently, according to the results of immunohistochemistry (IHC), breast cancer can be classified into Luminal A, Luminal B, HER2 over-expression, and triple-negative breast cancer (TNBC) (2). Based on HER2 expression breast cancer can be classified into HER2 positive (HER2 3+) and HER2 negative (HER2 1+, HER2 0). Patients with HER2 (2+) should undergo *in situ* hybridization (ISH) testing. Patients with positive ISH results are classified as HER2 positive, conversely, patients with negative ISH results are classified as HER2 negative. Of these, according to the latest data from the Surveillance, Epidemiology, and End Results Program (SEER) database, HER2-positive breast cancer accounts for about 14-15% of all breast cancer patients, which are highly aggressive and have a poor prognosis. The emergence of trastuzumab has led to a better prognosis for patients with HER2-positive breast cancer, which brings important implications for biologically targeted therapy for breast cancer (3). In recent years, it has been found that 45-60% of HER2-negative breast cancers should actually be classified as HER2 low-expression breast cancer. Whereas in previous classifications they were classified as Luminal and TNBC subtypes (4–6). There is no targeted treatment for patients with low HER2 expression, which leads to a less favorable prognosis for this group of patients. Classification of HER2 expression in breast cancer has been redefined by the advent of ADC drugs (7, 8). Whether HER2 low-expression can become a new subtype of breast cancer and whether HER2-expressing breast cancer can move from dichotomous (negative, positive) to trichotomous (negative, low-expression, positive) is the focus of attention.

## 2 Definition of HER2 low-expression breast cancer

Human epidermal growth factor receptor (HER) is often considered a negative prognostic factor for breast cancer. HER (HER1, HER2, HER3, and HER4) is located on human chromosome 17q21 and encodes a transmembrane tyrosine kinase receptor protein with a molecular weight of 185 KD. HER2 is a highly homologous protein with complex amino acid activity consisting of an extracellular lipophilic transmembrane region and an intracellular protein tyrosine kinase (PTK) active region group. The HER2 receptor is mainly located in the cell membrane and undergoes dimerization upon binding to epidermal growth factor (EFG) ligands. HER2 gene amplification in breast cancer is associated with increased cell proliferation, cell motility, tumor invasiveness, progressive regional and distant metastases, accelerated angiogenesis, and reduced apoptosis (9, 10). Breast cancer of different molecular types differ significantly in terms of clinicopathological features, therapeutic approaches, and prognosis. Recent studies have shown that novel ADC drugs targeting HER2 have strong activity in the treatment of breast cancer with HER2 low-expression (8). Based on the results of the clinical trials, while it is premature to create new result categories of HER2 expression, best practices to distinguish IHC 0 from 1+ are now clinically relevant. The new 2023 American Society of Clinical Oncology (ASCO), highlighted HER2 low-expression as patients with HER2 immunohistochemistry (IHC) (1+) or HER2 IHC (2+) and negative *in situ* hybridization (ISH) (11).

## 3 Characteristics of HER2 low-expression breast cancer

### 3.1 Clinical features of HER2 low-expression breast cancer

In clinical studies, the pathological features and prognosis of patients with different subtypes of breast cancer vary, and the results of different scholars on the clinicopathological features of HER2 low-expression breast cancer are also different. In a study by Onsum et al, it was found that the higher the IHC staining score of HER2-negative breast cancer, the higher the number of HER2 receptors that could be detected on the membrane of the cancer cells (12). HER2 receptors on the surface of breast cancer cells bind to ligands and promote tumor cell proliferation and invasion by activating signaling pathways. These results in breast cancers with HER2 low-expression having heavier tumor bodies and greater invasiveness than HER2 negative (HER2-0) breast cancer (13, 14). Ménard et al. found significant differences in the pathologic tissues of HER2 low-expression compared with HER2 negative patients (HER2-0). Among them, breast cancer patients with HER2 IHC2+ and negative ISH tests had larger tumor bodies, higher histological scores, more metastatic axillary lymph nodes and higher Ki-67 proliferation index (15). In the same study by Schettini et al, patients with HER2 low-expression were found to have higher T-stage, higher N-stage and higher histological grade compared with HER2-0 breast cancer, but there was no significant difference between the two groups in terms of Ki-67 proliferation index and the percentage of tumor-infiltrating lymphocytes (16). Currently, there are differences in the results of researchers’ analyses of the clinicopathological features of HER2 low-expression breast cancer (**Table 1**).

**Table 1:** Main clinical features of HER2 low-expression breast cancer %.

Variables	Ménard (15)					Schettini (16)		
	HER2 score				*P* value^a^	HER2 score		*P* value^a^
	HER2-0	HER-1+	HER-2+	HER-3+		HER2-0	HER2 low-expression	
T stage					0.201			0.007
T1	61.5	61.7	62.0	53.9		55.8	48.7	
T2	29.1	28.8	27,4	35.0		32.2	37.3	
T3	3.5	2.2	2.6	3.2		7.8	8.6	
T4	5.9	7.3	8.0	7.9		4.2	5.4	
N stage					<0.0001			0.01
+	47.3	63.7	56.8	59.1		41.2	44.4	
–	52.7	36.3	43.2	40.9		58.8	55.6	
Histological grades					<0.0001			0.0499
1	14.7	8.6	7.9	3.1		8.8	10.6	
2	55.3	50.4	50.1	33.1		35.6	39.1	
3	30.0	41.0	42.0	63.8		55.7	50.3	
Ki-67(Range)					<0.0001			0.092
<14%	43.6	31.8	31.4	15.3		43.9	38.9	
≥14%	56.4	68.2	68.6	84.7		56.1	61.1	

a Chi-squared P value calculated for evaluable data only.

### 3.2 Impact of hormone receptor status on clinical features of HER2 low-expression breast cancer

In addition, HR status is another important reference factor in the treatment of clinical breast cancer patients. Won et al. analyzed 30,491 cases of HER2-negative early-stage breast cancer and found that the clinical features of HER2 low-expression may be associated with HR status (17). Xinyang et al. collected data from 684 patients with primary HER2-negative breast cancer who underwent surgery and analyzed the clinicopathological characteristics of patients with HER2 low-expression breast cancer versus those with HER2-0. The results showed a higher proportion of HR-positive patients with HER2 low-expression breast cancer (approximately 89.6%) and a lower proportion of histological grade III cases in comparison with patients with HER2-0 breast cancer. Therefore, the difference in HR status makes a difference in the clinicopathologic characteristics of HER2-overexpression (HER2-3+) breast cancer (18). Yuyang Li et al. included 1,464 HER2-negative breast cancers associated with clinicopathologic features based on differences in HER2 expression and HR status. The results showed that more HR-positive breast cancer patients were premenopausal with HER2 low-expression breast cancer. They had fewer T4 tumors, higher histological grade, negative lymphovascular infiltration and higher human leukocyte antigen (HLA) expression (19). Pooled analysis of individual data from 2310 HER2-negative patients in four prospective neoadjuvant clinical trials by Denkert et al. It was found that the rate of pathological complete remission in the HR-positive subgroup of HER2 low-expression breast cancers was significantly lower than that of HER2-0 breast cancers (*p*=0.024) (**Table 2**), HER2 low-expression breast cancers were histologically more poorly graded and had lower Ki-67 proliferation indices (20). Schettini et al. showed that in HR-positive breast cancers classified by PAM50, the expression of ERBB2 and Luminal-related genes was significantly higher in the HER2 low-expression group than in the HER2-0 group (16). These data suggest that HR status is important for the clinical characterization of HER2 low-expression breast cancer. This may provide an important basis for the treatment of HER2 low-expression breast cancer (**Table 3**).

**Table 2:** Analyses of disease-free survival and overall survival by HER2 and hormone receptor status (%) (20).

		DFS (3-years)	*p*	OS (3-years)	*p*
**HR (+)**	HER2-low	82.8	0.39	92.3	0.13
	HER2-0	79.3		88.4	
**HR(-)**	HER2-low	84.5	0.0076	90.2	0.016
	HER2-0	74.4		84.3	

**Table 3:** Summary of drugs and the related clinical trials of HER2-low breast cancer.

Drugs	Clinical trials	Primary outcome	Results
ADC			
T-DM1	TDM4258g (37, 38)	ORR	In at least median HER2 expression patients: ORR = 42.9% In less than median HER2 expression patients: ORR = 38.2%
T-Dxd	NCT02564900 (8)	ORR, mPFS	ORR = 37% mPFS = 11.1 months
	DESTINY-Breast04 (43)	mPFS, OS	In HR+ patients: mPFS = 9.9 months OS = 23.4 months In HR− patients: mPFS = 8.5 months OS = 18.2 months
	DESTINY-Breast06	–	Ongoing
	DESTINY-Breast08	–	Ongoing
SYD985	NCT02277717 (7)	ORR, mPFS	In HR+ patients: ORR = 28% PFS = 4.9 months In HR− patients: ORR = 40% PFS = 4.1 months
Vaccine			
E75	NCT01479244 (50)	Disease recurrence	In E75 group: Disease recurrence = 54.1% In the placebo group: Disease recurrence = 29.2%
GP2	NCT00524277 (49, 51)	5-year DFS rate	In the vaccineeligible patients: The 5-year DFS rate = 88% In the control group: The 5-year DFS rate = 81%
AE37	NCT00524277 (51)	Disease recurrence 5-year DFS rate	In the immunized group: Disease recurrence rate = 12.4% 5-year DFS rate = 80.8% In the control group: Disease recurrence rate = 13.8% 5-year DFS rate = 79.5%
Bispecific antibody			
ZW25	NCT02892123	–	Ongoing
ZW49	NCT02892123	–	Ongoing

### 3.3 Prognosis of HER2 low-expression breast cancer

The expression of HER2 in breast cancer is markedly heterogeneous, mainly in terms of spatial heterogeneity and temporal heterogeneity among different cell subpopulations (4). Studies have shown that tumors with HER2 intra-tumor heterogeneity tend to have a poorer prognosis and are less sensitive to conventional anti-HER2 therapy (21, 22). A study by Tan et al. found that of 28,280 patients with non-metastatic HER2-negative breast cancer from six study centers, 43.4% were HER2 low-expression and 56.6% were HER2-0. In the HR-positive subgroup, recurrence-free survival and overall survival (OS) were significantly higher in the HER2 low-expression group than in the HER2-0 group (23). However, in another study analyzing data from 6477 patients from 12 studies, it was found that at a median follow-up of 90.3 months, differences in hormone receptor status did not have a significant effect on OS in either the HER2 low-expression group or the HER2-0 group (16). Results from a study from Germany (n=5907) found that patients with HR-positive HER2 2+ tumors had shorter median disease-free survival (DFS) (*p*<0.001). Analysis corrected for other prognostic factors found that HER2 2+ status was an unfavorable prognostic factor for DFS (HR=1.217, 95% CI: 1.052-1.408, *p*=0.008), but there was no significant difference between HER2 2+ and the other groups in HR-negative HER2-negative breast cancer patients with respect to DFS (24). The results of this experiment suggest that there is an effect of HR status on DFS in HER2 low-expression breast cancers. JuanJin et al. show that bone metastases are more common in breast cancer patients with HER2 low-expression types. The incidence of bone metastases in HER2-0 breast cancer was lowest in either the HR+ or HR- subtypes, both at the initial site of metastasis and during disease progression. And brain metastasis is a key marker of breast cancer prognosis. HER2-positive patients had the highest frequency of brain metastases in the total population. In the HR+ subgroup, the rate of brain metastasis was significantly higher in HER2 low-expression breast cancers than in HER2-0 breast cancers, and there may be differences in HER2 expression between primary and metastatic foci at different stages of progression. Low HER2 expression was seen in 38.1% and 28.8% of HER2-0 breast cancer patients in the primary tumor metastatic site in the overall population and HR subtype population, respectively. In contrast, 21.1% and 33.3% of HER2 low-expression primary tumors exhibited a HER2-0 phenotype in HR positive and HR negative metastatic tumors (25, 26). Therefore, the researchers concluded that the HER2 low-expression state is widely present in different stages or metastatic sites of HER2-positive breast cancer. The temporal and spatial heterogeneity of tumors leads to complication or even failure of the clinical treatment process. Therefore, the study suggests that clinicopathologic differences in HER2 low-expression breast cancers do not determine their prognosis. To date, studies on the characteristics of HER2 low-expression breast cancers have not achieved a consistent understanding.

## 4 Advances in the treatment of Her2 low-expression breast cancer

**Figure 1:**
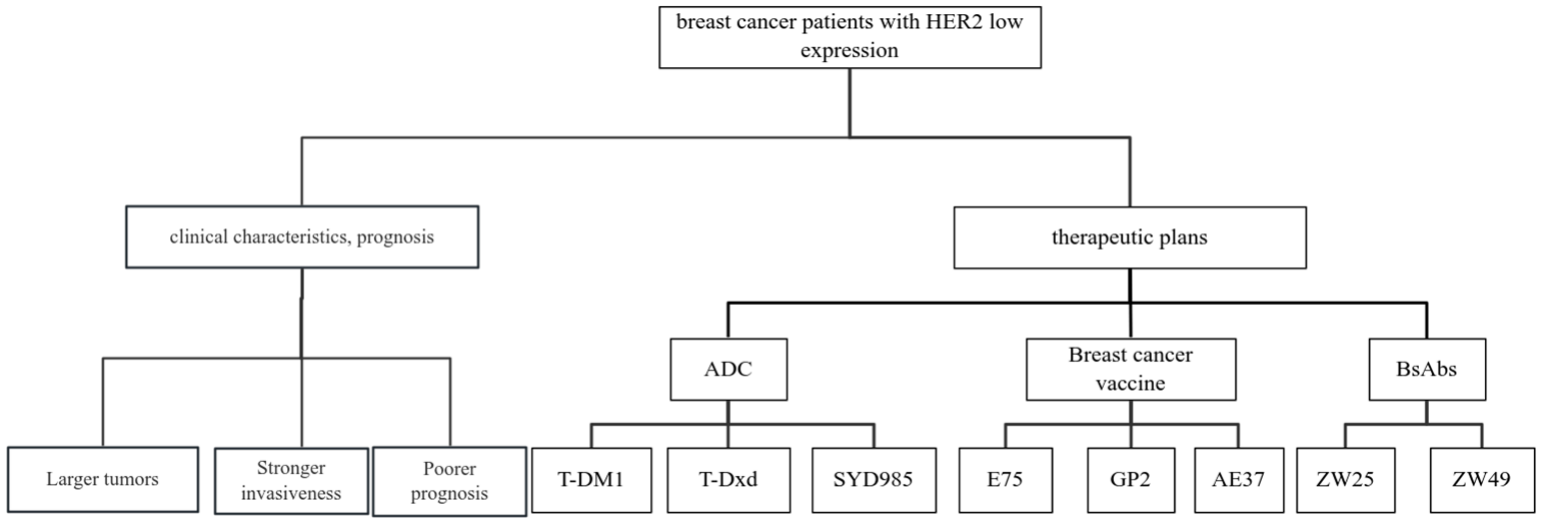
Review of clinical characteristics, prognosis and related treatment schemes of breast cancer patients with HER2 low expression.

HER2 is currently in the spotlight for researchers in targeted breast cancer therapies (27). There are currently three main classes of HER2-targeted therapies approved for marketing: the monoclonal antibodies (trastuzumab and patuximab), the oral tyrosine kinase inhibitors (lapatinib, neratinib, pyrrolitinib and tucatinib), and the ADC (T-DM1 and T-Dxd). These anti-HER2 therapeutic agents play an important role in neoadjuvant, postoperative adjuvant, and late-stage salvage therapy for patients with HER2 over-expression breast cancers. However, due to low Her2 expression, patients with HER2 low-expression breast cancer are insensitive to conventional anti-Her2 targeted drug therapy. HER2-targeted therapies have long been available for patients with HER2-positive breast cancers. However, with the discovery of emerging agents such as T-DM1, T-Dxd, and SYD985, patients with HER2 low-expression breast cancers may also benefit. Breast cancer vaccines are also being explored for use in the treatment of HER2 low-expression breast cancers. The use of anti-HER2 therapies in HER2 low-expression breast cancer has also received attention from researchers. Other mechanisms are being investigated, and the following is a detailed description of the drugs that are currently being investigated and are expected to be used in the treatment of patients with clinical HER2 low-expression breast cancer (**Figure 1**).

### 4.1 ADC

Novel ADC findings lead to important breakthroughs in anti-HER2 therapy. Its pharmacological mechanism does not depend on the high HER2 expression status, and it also has good efficacy in patients with low HER2 expression. ADC drugs typically contain three key components: antibodies with high specificity and affinity, highly stable linkers and potent small molecule cytotoxicity (28, 29). After the ADC drug enters the bloodstream, the antibody component recognizes the target and binds to tumor cells that highly express the antigen on the cell surface. The ADC antigen complex enters the tumor cell by endocytosis. Lysosomal degradation releases a cytotoxic load (an anti-dividing agent) that destroys DNA or prevents tumor cells from dividing, killing the cells (30). Therefore, ADC drugs combine the advantages of strong monoclonal antibody targeting and high small molecule cytotoxic activity. ADC reduces the systemic toxicity of small molecule cellular drugs and improves anti-tumor efficacy. In addition to the anti-tumor effect of specific antibodies and the killing effect of cytotoxic drugs internalized by tumor cells, there is another important principle in the killing of ADC drugs: the bystander effect. The bystander effect refers to the fact that cytotoxic drugs are internalized and degraded by ADC and then released from the target cell, or taken up by surrounding tumor cells in the extracellular space and effectively killed. These cells themselves may or may not express ADC target antigens. Breast cancer cells with low HER2 expression express 50-1 million HER2 receptor molecules on their membranes, which provides the conditions for the use of ADC drugs in targeted therapy (31, 32). An important factor affecting the efficacy of ADC is the antibody ratio (DAR). It reflects the average number of chemotherapeutic drugs linked to each antibody and also affects the stability, efficacy and toxicity of the drug in the body’s circulation (33).

#### 4.1.1 T-DM1

T-DM1 is the first approved ADC drug for breast cancer. T-DM1 consists of trastuzumab, the maytansinoid derivative DM1, and a noncleavable thioether linker called N-maleimidomethyl cyclohexane-1-carboxylate (MCC) (34). Each antibody is coupled to an average of 3.5 DM1s, which are covalently coupled to the lysine residues of trastuzumab (DAR=3.5). The mechanism of action of DM1 is to bind to microtubule proteins and inhibit their polymerisation to induce cell cycle arrest and apoptosis. T-DM1 binds to HER2 allowing the complex to enter target cells via receptor-mediated endocytosis. The antibody component of T-DM1 is degraded in lysosomes and DM1 is released into the cytoplasm, leading to cell cycle arrest and induction of apoptosis (35, 36). However, the therapeutic effect of T-DM1 against HER2 low-expression breast cancer is not satisfactory. Two phase II trials have investigated the efficacy and safety of T-DM1 in patients with HER2-positive metastatic breast cancer previously treated with trastuzumab. T-DM1 was found to have poorer clinical activity in patients with HER2 low-expression and HER2-0 breast cancer compared to patients with HER2-positive breast cancer (37, 38). The possible reasons for the lack of efficacy of T-DM1 in patients with HER2 low-expression are twofold: (1) T-DM1 is a potent anti-microtubule protein Medenin derivative DM1 conjugated to trastuzumab via a non-cleavable thioether junction. The uncut connector requires hydrolysis within the cellular lysosome for potency, which makes cleavage and re-release of cytotoxic drugs after entry into the tumor cell difficult and lacks a bystander effect (39). (2) The drug-antibody ratio of T-DM1 is only 3.5:1, and the cytotoxicity of the drug that reaches the tumor cells is low, so the killing effect is limited (40).

#### 4.1.2 T-Dxd

Trastuzumab-deruxtecan (T-Dxd) is the world’s first ADC drug to show a survival benefit in people with HER2 low-expression. T-DXd has a higher DAR (≈8:1) and a novel cytotoxic load compared to T-DM1. T-Dxd is a novel HER2-targeted ADC. It consists of a humanized anti-HER2 antibody, an enzymatically cleavable peptide linker and a novel topoisomeraseI (TOPOI) inhibitor. The anti-HER2 antibody portion of T-Dxd is a human monoclonal IgG1 produced with reference to the same amino acid sequence as trastuzumab (41). TOPOI inhibitor has potent tumor killing effect and good cell membrane permeability. It interferes with DNA replication and transcription by capturing the TOPOI cleavage complex and exerts greater cytotoxic activity at a lower dose (42). The good membrane permeability allows the load to kill the tumor cells that contain it, as well as cross the cell membranes of neighboring tumor cells, thus killing the tumor tissue more effectively. Fifty-four patients with advanced HER2 low-expression breast cancer enrolled in study NCT02564900 received ≥1 dose of T-Dxd at either 5.4 (21 patients) or 6.4 mg/kg (33 patients). The median number of prior lines of therapy was 7.5 in 54 subjects, 83.3% had experienced ≥5 prior lines of therapy, and HER2 expression was IHC 0 in 5 (9.3%) patients, IHC 1+ in 30 (55.6%), and IHC 2+ in 14 (25.9%). The overall response rate (ORR) was found to be 37.0% (95% CI: 24.3% ~ 51.3%) and the median time to response was 10.4 months (95% CI: 8.8 months ~ unable to assess). T-Dxd exhibits preliminary anti-tumor activity in HER2 low-expression breast cancer patients (8). DESTINY-Breast04 is a study designed to investigate the efficacy of T-Dxd in HR-positive, HER2 low-expression advanced breast cancer. Findings published by the American Society of Clinical Oncology (ASCO) 2022 indicated that in the HR-positive population, there was a significant benefit in mPFS in the T-Dxd group compared to the treatment of physician’s choice (TPC) group (10.1 months vs. 5.4 months, HR=0.51, *p*<0.0001). In the total population, the mPFS in the T-Dxd group had the same benefit outcome (9.9 months vs. 5.1 months, HR=0.50, *p*<0.000 1). In terms of overall survival (OS), in the HR-positive population, the T-Dxd group was prolonged by 6.4 months compared with the TPC group (23.9 months vs. 17.5 months, HR=0.64, *p*<0.0028). The OS in the T-Dxd and TPC groups in the total population was 23.4 and 6.8 months, respectively (HR=0.64, *p*<0.0010). This is the first phase III trial to achieve positive results in breast cancer patients with low HER2 expression and the first phase III trial to be conducted in breast cancer patients with HER2 low-expression (43). Currently, several clinical trials on T-Dxd continue to be explored. The ongoing DESTINY-Breast06 study has further refined the categorization of HER-2 low-expression. This trial will further explore the anti-tumor effects of T-Dxd in patients with very low HER2 expression (44). Another phase Ib trial, DESTINY-Breast08, is exploring the safe dosage of T-Dxd in combination with other drugs in patients with HR+, HER-2 low-expression advanced breast cancer. We look forward to the publication of the results of these studies, which will provide more rationale for targeted therapy in HER2 low phenotype breast cancer.

#### 4.1.3 SYD985

Trastuzumab-Duocarmazine (SYD-985) is a novel ADC drug in the early stages of clinical development. It consists of trastuzumab, a cleavable junction and seco-DUBA (DAR=2.8:1) (45). SYD985 cytotoxic drug fraction seco-DUBA is a prodrug that penetrates cell membranes. Upon binding of SYD985 to HER2, seco-DUBA is internalized by tumor cells, and the prodrug is cleaved by proteases in the lysosome to dorcamycin, which alkylates DNA. Prodrugs can also be cleaved in the interstitium by proteases secreted by tumor cells, thus becoming active cytotoxic drugs with bystander effects (46). Due to its bystander effect, SYD-985 has shown some efficacy in a cellular model of HER-2 over-expression breast cancer, although the drug-antibody ratio is relatively low (45). SYD985 is 3- to 50-fold more cytotoxic than T-DM1 in HER2 over-expression cell lines. In a patient-derived breast cancer xenograft model with low HER2 expression, SYD985 showed strong anti-tumor activity, while T-DM1 was inactive (47). A phase I trial of SYD-985 was conducted on HER2 low-expression breast cancer, and the results showed that SYD-985 had good clinical activity against HER2 low-expression breast cancer. In this trial, all patients had low HER2 expression, with 9 of 32 HR+ breast cancer patients (28%, 95% CI 13.8-46.8%) achieving objective remission, and 6 of 15 HR- breast cancer patients (40%, 95% CI 16.3-67.6%) achieving objective remission. The mPFS was 4.1 (95% CI 2.4-5.4) months for HR+ breast cancer patients and 4.9 (95% CI 1.2-N/E) months for HR- breast cancer patients (7).

### 4.2 Breast cancer vaccine

Vaccines for breast cancer treatment include peptide vaccines, protein vaccines, nucleic acid vaccines (DNA/RNA vaccines), bacterial/viral vaccines and different immune cell vaccines. When used alone or in combination with other immunotherapies, they have become an attractive class of cancer immunotherapy. Employing the immune system to eliminate breast cancer cells is a novel therapeutic modality (48). In breast cancer, the most studied tumor-associated antigen (TAA) is HER2, and several peptides derived from the HER2 protein have been shown to elicit immune responses (49). One such peptide is E75 (nelipepimut-S) which, in a phase I/II clinical trial vaccinating breast cancer patients in the adjuvant setting to prevent disease recurrence, was found to have a five-year disease-free survival (DFS) rate of 90% in vaccinated patients versus 80% in unvaccinated control patients (50). GP2 is a HER2-derived, HLA-A2+ restricted peptide. NCT00524277 enrolled HLA-A2+, clinically disease-free, node-positive and high-risk node-negative breast cancer patients with tumors expressing HER2 (IHC 1+-3+). The study found that among HER2+ patients, the 5-year DFS rate was estimated to be 94% (82%-98%) in vaccinated patients (n = 51) compared with 89% (71-96%) in control patients (n = 50) (*p* = 0.86) (49). AE37 is the Ii-Key hybrid of the MHC class II peptide, AE36 (HER2 aa:776–790). The results confirm that for IHC 1+ or 2+ HER2-expressing tumors, the estimated 5-year DFS was 77.2% in vaccinated patients (n = 76) versus 65.7% in control patients (n = 78) (HR.595,95% CI 0.263–1.347, *p* = 0.21) (51). Due to the specificity of the vaccine, most of the research on vaccines has been limited to postoperative prophylaxis of early-stage breast cancer. Most patients with HER2 (1+-3+) develop an immune response, and those with low HER2 expression have a stronger immune response and may derive greater clinical benefit from some vaccines.

### 4.3 Bispecific antibodies

BsAbs are engineered molecules designed to bind two antigens simultaneously. This feature targets TAAs on breast cancer cells while engaging immune effector cells or blocking critical signaling pathways. By harnessing this dual targeting ability, BsAbs can enhance tumor specificity and induce robust immune responses against breast cancer cells (52). Bispecific antibodies have a more efficient mechanism and more stable binding than monoclonal antibodies. Zanidatamab (ZW25) is a novel bispecific antibody against HER2 that targets both the classical ECD4 epitope (trastuzumab binding domain) and the ECD2 epitope (patuximab binding domain) (53). Each arm of the NCT02892123 trial included a subset of breast cancer patients with low HER2 expression. In this trial, ZW25 showed good tolerability and good anti-tumor activity. This implies that ZW25 has great potential for the treatment of HER2-overexpressing breast cancer (54). Similar to ZW25 is ZW49, for which research is in progress.

## 5 Discussion

HER2 low-expression breast cancers account for about half of all subtypes and are gaining importance. Previously, HER2 low-expression breast cancers often do not benefit from conventional targeted therapies due to insufficient receptor expression. In terms of clinical features, the HER2 low-expression status is distinct from HER2 over-expression and HER2-negative breast cancer. HER2 low-expression is a specific HER2 expression state, which can also be interpreted as a state of heterogeneous HER2 expression in breast cancer. Current findings suggest HER2-targeted therapy may benefit patients with HER2 low-expression breast cancer. The prognosis of the HER2 low-expression breast cancer population has been greatly improved by the publication of promising clinical data on new ADC drugs represented by T-Dxd. HER2 low-expression breast cancer is gaining attention due to its unique clinical biological features. Future targeted therapeutic strategies for HER2 low-expression breast cancer will focus on the following improvements to the ADC class of drugs: (1) Modifying linker drugs and linker structures to maximise bystander effects; (2) Increasing the load of anti-personnel drugs; (3) Introducing double or even multiple resistances, etc. It is believed that the development of the ADC class of drugs for HER2 low-expression breast cancer will bring revolutionary advances in the diagnosis and treatment of this type of breast cancer. Clinical trials of other treatments such as bispecific antibodies and breast cancer vaccines are clinical trials are also underway, and we look forward to subsequent results.
